# Effects of Glucose Tablet Candy Ingestion on Attention Following Smartphone Use in Healthy Adults: A Randomized, Double-Blind, Placebo-Controlled Crossover Trial

**DOI:** 10.3390/foods14244233

**Published:** 2025-12-09

**Authors:** Yuko Setoguchi, Motoki Tsukiashi, Hiroko Maruki-Uchida, Naoki Iemoto, Shukuko Ebihara, Takashi Mato

**Affiliations:** 1 R&D Institute, Morinaga & Co., Ltd., 2-1-1 Shimosueyoshi, Tsurumi-ku, Yokohama 230-8504, Japan; m-tsukiashi-ai@morinaga.co.jp (M.T.); h-uchida-ji@morinaga.co.jp (H.M.-U.); n-iemoto-jb@morinaga.co.jp (N.I.); 2 Chiyoda Paramedical Care Clinic, 3-3-10 Nihonbashi Hongokucho, Chuo-ku, Tokyo 103-0021, Japan; s.e@cpcc.co.jp; 3 Department of Emergency and Critical Care Medicine, Jichi Medical University, 3311-1 Yakushiji, Shimotsuke 329-0498, Japan; mato_stm@jichi.ac.jp

**Keywords:** glucose, attention, concentration, cognitive function, smartphone

## Abstract

**Background/Objectives**: Excessive smartphone use may negatively affect cognitive functions, including attention. While sensorimotor rhythm, beta, and theta waves have been linked to concentration, the electroencephalography (EEG) frequency band that most reliably serves as a neurophysiological marker of concentration is unclear. Therefore, we aimed to evaluate the effects of glucose tablet candy ingestion on attention following smartphone use in healthy adults. **Methods**: A randomized, double-blind, placebo-controlled crossover trial was conducted in 16 healthy adults aged 18–39 years. Participants performed a 30 min smartphone-based information search task. Attention was assessed before and after the task using the Cognitrax test battery, and participants ingested either a glucose tablet candy (containing 26 g of glucose) or a placebo (no glucose) between tests. EEG was performed during attention tests using a patch-type device. Subjective sensations, including attention, fatigue, and mental clarity (clear-headedness), were evaluated using a visual analog scale (VAS). The primary outcome was attention test scores, and secondary outcomes included EEG power and VAS ratings. **Results**: Glucose tablet candy ingestion after smartphone use significantly improved mean correct response time and error response scores in part 2 of the four-part continuous performance test, a subtest within Cognitrax, compared to that with the placebo. Additionally, glucose intake significantly attenuated the decrease in right prefrontal beta EEG power observed with the placebo. Improvements were also observed in self-reported physical fatigue and mental clarity on the VAS following glucose ingestion. **Conclusions**: The ingestion of the glucose (26 g) tablet candy improved sustained attention after smartphone use in healthy adults aged 18–39 years and was associated with changes in brain activity. These results suggest that the glucose tablet candy may help counteract the decline in concentration following cognitively demanding smartphone use.

## 1. Introduction

The use of smartphones has increased worldwide. In Japan, the penetration rate was 97.5% in 2023 [1]. Consequently, smartphones have become the primary device for Internet access. The average daily smartphone Internet usage time on holidays is 151.4 min overall, as high as 291.0 min among teenagers, and 244.2 min among individuals in their twenties. Concerns have been raised regarding the adverse effects of excessive smartphone and Internet use, which have negative impacts on academic performance, learning abilities, and cognitive functions [2,3]. A longitudinal study tracking 223 children (aged 8.4–21.3 years) over 3 years revealed that higher frequency of Internet use was associated with decreases in verbal intelligence quotient and gray matter volume in the brain [4]. Long- and short-term effects have been reported, such as decreased reaction speed in divided attention tasks after 15 min of online shopping [5] and increased error rates in sustained attention tasks after 30 min of computer gaming [6]. Furthermore, trials using smartphones have reported delayed psychomotor vigilance task response times and increased error rates in Go/NoGo tasks following 45 min of social networks (SNS) or Internet searching [7], as well as reduced Stroop test accuracy and slower reaction times after 30 min of SNS or video gaming [8].

Glucose is the primary energy source for the healthy human brain, accounting for approximately 25% of the total body glucose consumption at rest [9]. However, the brain cannot store glucose and relies on a continuous supply of approximately 120 g/day via the bloodstream [10,11]. Many studies have investigated the impact of orally ingested glucose on cognitive function, demonstrating its efficacy in improving verbal memory, visuospatial memory, episodic memory, long-term memory, working memory, executive function, problem solving, attention, and face recognition [9,11,12]. In our previous studies, we demonstrated that ingestion of a tablet candy (pressed candy) containing 26 g of glucose led to improvements in working memory and sustained attention, as assessed by Cognitrax (Health Solution Inc., Tokyo, Japan) [13]; improved selective attention accompanied by increased brain activation, as measured by functional near-infrared spectroscopy [14]; and enhanced information processing speed and sustained attention after playing esports [15]. Although the beneficial effects of glucose tablet candy on several cognitive functions, including attention and sustained concentration, have been demonstrated, it remains unclear whether they extend to situations where attention is temporarily impaired by cognitively demanding smartphone use.

In our previous study, where we evaluated cognitive function after playing esports, a portable electroencephalography (EEG) device revealed an increased proportion of sensorimotor rhythm (SMR) power during gameplay, suggesting that glucose intake may be beneficial for maintaining concentration [15]. While SMR [15,16], beta [16,17], and theta waves [18,19] have been linked to concentration depending on the EEG device, electrode placement, and research methodology, neurophysiological markers of concentration have not yet been fully elucidated.

Here, we aimed to investigate whether glucose-containing tablet candy can mitigate attention deficits induced by cognitively demanding smartphone use in healthy adults aged 18–39 years, using a randomized, double-blind, placebo-controlled, crossover design. Concentration (attention) was evaluated using an attention task with concomitant observation of EEG changes across a wide range of frequency bands to examine the effects of glucose intake. In this study, we selected a 30 min smartphone information search task to induce cognitive load, as previous studies have shown that similar task types and durations can result in attentional impairment and cognitive fatigue [5,6,7,8]. Accordingly, our study reflects real-world situations in which prolonged smartphone use may negatively affect attention and assesses whether acute glucose tablet candy supplementation can counteract these effects.

## 2. Materials and Methods

### 2.1. Study Design and Participants

**Figure 1:**
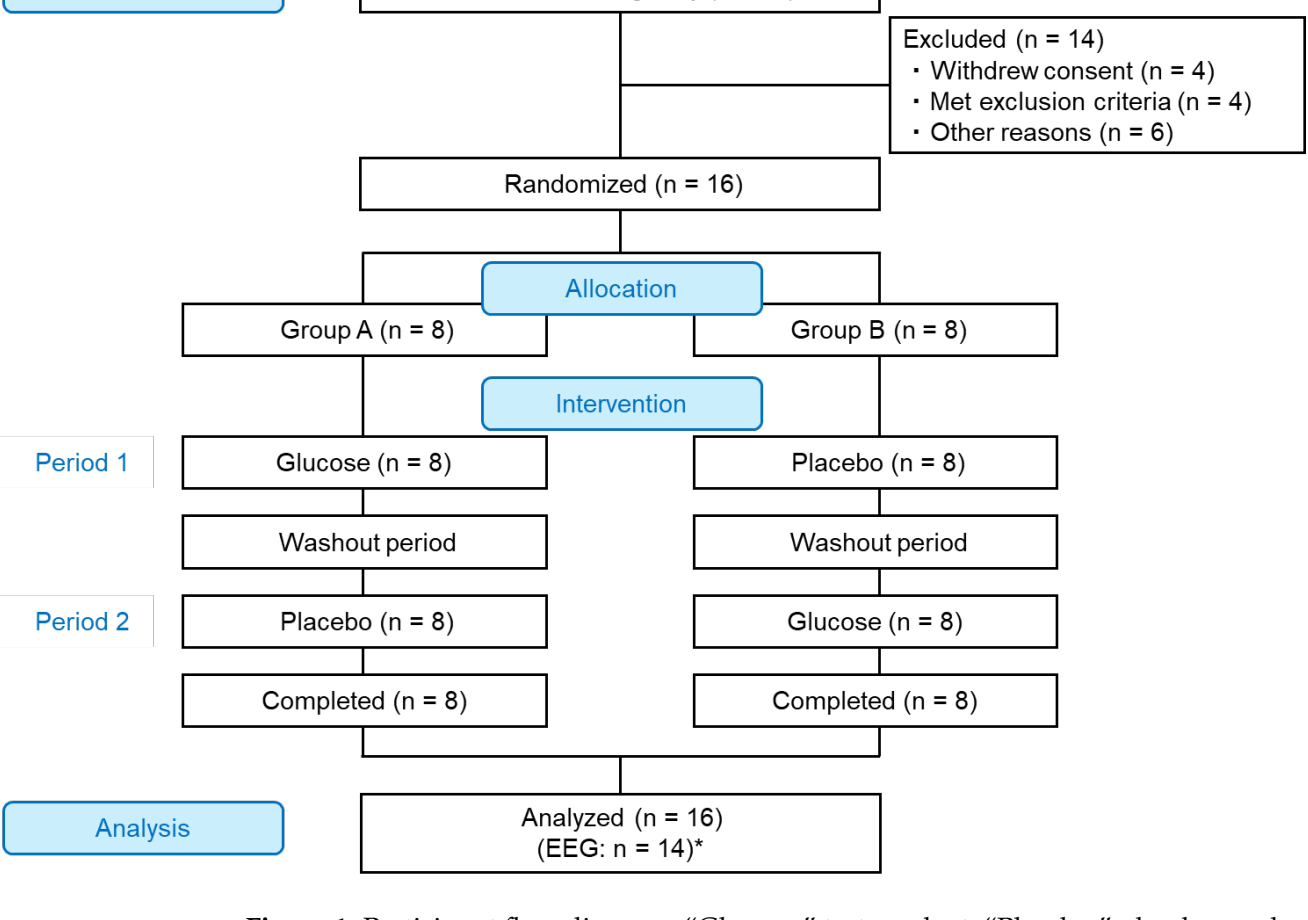
Participant flow diagram. “Glucose,” test product; “Placebo,” placebo product. * Electroencephalography (EEG): *n* = 8 in Group A; *n* = 6 in Group B for technical reasons.

A randomized, double-blind, placebo-controlled crossover trial was conducted at the Chiyoda Paramedical Care Clinic (Tokyo, Japan) from November 4 to December 9, 2024. Sixteen healthy men and women aged 18–39 years (Table 1) were recruited through advertisements and screened according to predefined inclusion and exclusion criteria (Table S1). The sample size was calculated using the results of sustained attention from our previous crossover trial using Cognitrax in adults aged 20–39 years [13]. Assuming a difference in means between groups of 8.6 and standard deviation of 10.2, with a statistical power of 0.8 and an alpha error of 0.05, the required sample size was estimated to be 14. To account for possible dropouts, the target enrollment was set at 16 participants. All the participants underwent two test periods, receiving both the test and placebo treatments in a randomly assigned order (crossover). Each period was conducted on the same weekday and at the same time of day for each participant, with washout period of at least 6 days between periods (Figure 1).

Ethical approval was obtained from the Chiyoda Paramedical Care Clinic Ethics Committee (approval date: 20 September 2024). This study was registered with the University Hospital Medical Information Network (UMIN) Clinical Trials Registry (No. UMIN000055810). All procedures were conducted in accordance with the ethical principles for medical research involving human participants set forth by the World Medical Association (Declaration of Helsinki) and Ethical Guidelines for Life Sciences and Medical Research Involving Human Subjects issued by the Ministry of Education, Culture, Sports, Science and Technology; Ministry of Health, Labour and Welfare; and Ministry of Economy, Trade and Industry, Japan. All participants provided written informed consent prior to enrollment.

### 2.2. Test Product

The test product was a commercially available glucose tablet candy (“Ramune,” Morinaga & Co., Ltd., Tokyo, Japan), with each package weighing 29 g and supplying 26 g glucose. The placebo tablet candy (primarily erythritol-based, 29 g) matched in flavor and appearance to the test product, making it indistinguishable to participants. Each product was packaged in an identical container and distributed to the participants immediately before ingestion. The nutritional content per package was as follows: The test product contained 108 kcal energy, 26.3 g carbohydrates, 0.34 g fat, and 0 g protein; the placebo product contained 8.8 kcal energy, 28.4 g carbohydrates, 0.34 g fat, and 0 g protein. The glucose dose (26 g) was selected to be consistent with the amounts used in our previous related studies examining cognitive and neurophysiological effects [1,15].

### 2.3. Study Procedure

#### 2.3.1. Screening and Familiarization

After obtaining written informed consent, 16 participants were selected from the 30 initially screened individuals who underwent preliminary testing. The 16 participants were randomly assigned to 2 groups of eight each, with the order of test product and placebo intake counterbalanced between the groups (Figure 1). Group allocation ensured minimal differences in age, sex, and baseline Cognitrax scores between groups at the time of screening. The allocation sequence was password-protected and securely stored by the allocation manager, and the group assignments were blinded to the participants, investigators, and data analysts throughout the study until all data were finalized.

For the screening visit, the participants were instructed to refrain from consuming any food or drink, except water, for at least 4 h prior to arrival. Participants then underwent a medical interview by a physician, anthropometric measurements (height, weight, and BMI), and physiological assessments (systolic and diastolic blood pressure, and pulse rate). An attention test was then conducted to allow participants to familiarize themselves with the test procedure. Subsequently, an EEG device was fitted with each participant to confirm accurate signal acquisition under the same environmental conditions as those in the main trial.

#### 2.3.2. Intervention Procedures

The participants received both test products (glucose and placebo) in a randomized order across the two periods. In this section, and subsequent tables and figures, “glucose” and “placebo” denote the respective interventions.

For each main trial period, the participants were instructed to refrain from consuming any food or beverage, except water, for at least 4 h prior to arrival, as in the screening phase. Upon arrival, the participants underwent a medical interview with a physician, body weight measurements, and physiological assessments (systolic and diastolic blood pressure, and pulse rate). A visual analog scale (VAS) was used. The participants were fitted with an EEG device and a 2 min resting-state EEG was recorded in a seated position.

Next, participants completed an approximately 20 min attention test (pre-attention test), during which, EEG data were simultaneously collected.

After completing the pre-attention test and VAS, the participants were provided with a smartphone, task sheet, and answer sheet. They were instructed to perform an Internet information search for 30 min according to standardized task instructions. They then briefly recorded their results using keywords on the answer sheet. Participants were specifically encouraged to focus on information gathering, spending minimal time on writing. If they could not complete all items within the allotted time, incomplete answers were acceptable. If they finished early, they were asked to continue searching and refining their responses until the end of the task. The assigned tasks were designed to simulate real-world cognitive demands commonly encountered when searching for complex information with a smartphone. For example, one assignment required participants to plan the launch of a foreign-themed cafe, selecting features, such as menu items, interior design, and store name based on online research. Both tasks were unfamiliar to participants and involved multiple steps and decision making under time pressure. Detailed content of the tasks is not provided in the present manuscript so as to minimize bias and maintain generalizability.

After completing the information-search task, the participants completed another VAS. They were then provided with the allocated test product and water, and instructed to consume the test product within 5 min. The test product was consumed with the water provided as needed. After ingesting the test product, the participants were asked to drink 100 mL of water to ensure that no residue remained in the oral cavity.

Subsequently, they received the same answer sheet used during the information search. They were asked to recall and write down as much information as they could easily remember.

**Figure 2:**
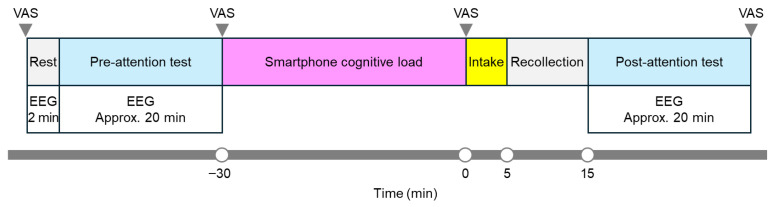
Protocol timeline. EEG, electroencephalography.

Fifteen minutes after starting to consume the test product, the participants again completed the attention test (post-attention test), during which EEG data were simultaneously collected. Following the completion of the post-attention test, another VAS was administered. The timeline for each test day is shown in Figure 2.

### 2.4. Efficacy Assessment Measures

#### 2.4.1. Primary Outcome: Attention Test

Cognitive performance was assessed using the Cognitrax (Health Solutions Inc., Shibuya-ku, Tokyo, Japan) [20]. The test battery included the Stroop test (ST), shifting attention test (SAT), continuous performance test (CPT), and four-part continuous performance test (FPCPT). Standardized scores for each parameter were used for the evaluation. The standardized score is a converted value calculated using the mean for the same age group set at 100 and standard deviation of 15. Details for each test are provided in Table S2.

#### 2.4.2. EEG

EEG were recorded using a patch-type device, HARU-2 (PGV Inc., Chuo-ku, Tokyo, Japan). HARU-2 is lightweight, wireless, and designed with an elastic electrode sheet to minimize the physical burden during attachment. The electrodes were positioned on the sheet at the mid- (ChZ), right (ChR), and left (ChL) forehead. The device was attached to the participant’s forehead to avoid contact with hair and the reference electrode was placed on the mastoid behind the left ear.

The preprocessing of EEG data and calculation of frequency power values were outsourced from PGV Inc. EEG preprocessing was performed according to the methods specified by PGV Inc.: a 0.5–90 Hz band-pass filter was applied as requested to enable analysis across a wide frequency range, and a 50 Hz notch filter, a 400-μV artifact rejection threshold, and Hamming window processing (2 s window, 50% overlap) were implemented as specified by PGV. Data segments were then subjected to fast Fourier transformation for power spectra. Frequency bands were defined as follows: delta (0.5–3.5 Hz), theta (4–7.5 Hz), alpha (8–12.5 Hz), beta (13–29.5 Hz), low gamma (30–49.5 Hz), and high gamma (50–70 Hz). The power values for each frequency band were calculated in 1 s epochs.

For each power value (X) in each frequency band, the mean (μ) and standard deviation (σ) were calculated across all recording periods (resting, pre-attention test, and post-attention test) for each participant on each test day, and Z-scores were computed as Z = (X − μ)/σ. Mean Z-score values were then calculated for the resting state as well as for the first and second halves of both the pre- and post-attention tests, and these were used for analysis.

#### 2.4.3. VAS

Subjective sensations were assessed using the VAS. At each time point, participants were asked to rate six subjective feelings, concentration, physical fatigue, mental fatigue, mental clarity (clear-headedness), motivation, and sleepiness, by marking a point on a 10 cm line with “best feeling” at the left end and “worst feeling” at the right end.

### 2.5. Statistical Analysis

All efficacy endpoints were analyzed using a linear mixed model with a two-sided significance level of 5% and were implemented using IBM SPSS Statistics 30 (IBM Japan, Tokyo, Japan). To evaluate the effects of the test product, the outcome variables were set as standardized post-attention test scores, EEG power value Z-scores during the post-attention test, and VAS scores after the post-attention test. The fixed effects included product (glucose vs. placebo), sequence (Group A vs. Group B), and intervention period (period 1 vs. period 2), whereas a unique patient identifier was included as a random effect. For covariate adjustment, pre-attention test values were used as baseline covariates for the analyses of post-attention test scores and EEG parameters, and post-smartphone cognitive load VAS scores were used as baseline covariates for the analyses of subsequent VAS scores.

For the evaluation of the effects of each task (pre-attention test and smartphone cognitive load), the outcome variable was the VAS score measured at the end of each task. The fixed effects included measurement point (pre-task vs. post-task), sequence, and intervention period, with a unique patient identifier as a random effect.

For EEG data that could not be reliably acquired, missing data imputation was not performed; these cases were treated as missing values only for the respective endpoint, whereas data from other evaluable endpoints for those participants were included in the analysis.

## 3. Results

### 3.1. Participants

All the participants enrolled in this study completed the trial; thus, 16 were included in the analysis (Figure 1). There were no missing data in the attention tests or VAS questionnaires; therefore, all data for these measures were included in the analyses. For the EEG, one participant who failed to provide a signal during the post-attention test in period 1 and another participant whose data in period 2 contained excessive movement artifacts in more than half of the measurements were excluded from the EEG analysis. For each participant, EEG data from both periods were excluded from the analysis, regardless of the data quality in the other period. Both the excluded participants belonged to Group B. No adverse events were reported during the study period.

### 3.2. Attention Test

The standardized scores for the pre- and post-attention tests are presented in Table 2. After glucose consumption, the mean standardized scores for the average correct response time and incorrect responses in the FPCPT2 group were significantly higher than those in the placebo group. No significant effects of period or sequence were observed. FPCPT2 is a sustained attention task in which various combinations of colors and shapes are randomly presented on the screen. Participants were instructed to quickly press a key when a green circle appears. For other attention test indices following the consumption of glucose or placebo, no significant differences were observed between the conditions, although some indices in the ST and SAT groups showed significant period effects.

### 3.3. EEG

**Figure 3:**
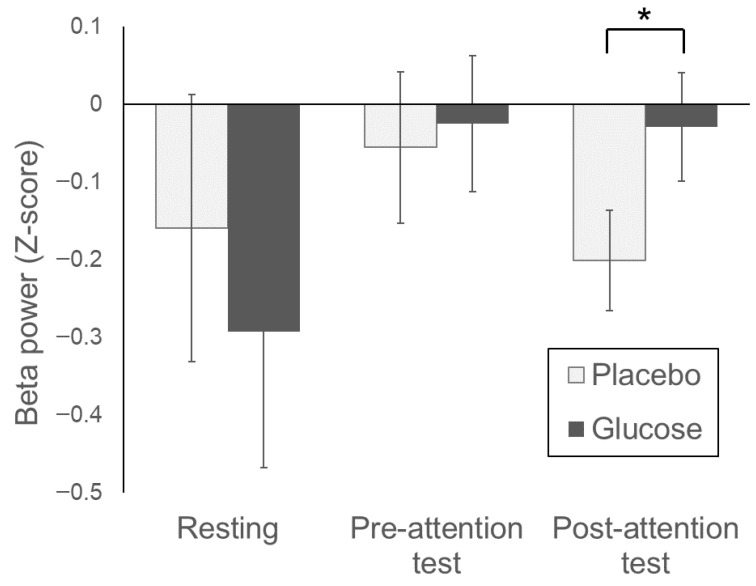
Beta power (Z-score) at the right prefrontal electrode. Bars indicate mean ± standard error (SE). Statistical significance (* *p* < 0.05, Glucose vs. Placebo) was determined using a linear mixed model.

For the mean Z-score of beta power at the ChR, an increase was observed from the resting state to the pre-attention test in the placebo condition; however, this value returned to baseline (resting) levels after the post-attention test (Figure 3). In contrast, in the glucose condition, the elevated beta power persisted even during the post-attention test and decline observed with the placebo was significantly suppressed. No significant period or sequence effects were observed. No significant effects of the condition were detected for the other frequency bands and electrodes, nor were there any significant period or sequence effects.

EEG power was analyzed for all recorded frequency bands (delta, theta, alpha, beta, low gamma, and high gamma) at each of the three frontal channels (ChL, ChZ, and ChR; Figure S1). For all frequency bands and electrodes, except beta power at ChR, no significant glucose intake, period, or sequence effects were observed.

Additionally, Figure S1 shows the following tendencies: in alpha, beta, low gamma, and high gamma bands, power tended to increase during the attention tasks (pre- and post-attention tests) compared with those in the resting state across multiple channels. Conversely, theta band power tended to decrease during the attention tasks compared with that in the resting state.

### 3.4. VAS

Physical fatigue and mental clarity (clear-headedness) after the post-attention test were significantly improved following glucose intake compared with that with the placebo (Table 3). No significant effects of period or sequence were observed.

To investigate the effects of smartphone cognitive load, VAS scores after the cognitive load task (post-load) were compared with those after the pre-attention test (pre-load). Significant improvements were observed in VAS scores for concentration, mental clarity, and sleepiness (Table 4). A significant effect of period was observed on sleepiness. Additionally, for reference, changes in the VAS scores from arrival to after the pre-attention test were compared. The pre-attention test resulted in significant increases in VAS scores for concentration, physical fatigue, mental fatigue, mental clarity, and motivation. No significant period or sequence effects were observed.

## 4. Discussion

In this study, we investigated the effects of a single ingestion of a commercially available tablet candy containing 26 g of glucose on attention and EEG activity following a cognitively demanding smartphone cognitive load in healthy adults aged 18 to 39 years, using a randomized, double-blind, placebo-controlled crossover design. Both test products were indistinguishable in appearance, texture, and flavor, and provided in identical containers on each trial day, minimizing expectation bias and permitting an objective assessment of the effects associated with glucose intake.

Notably, unlike our previous work, the present study specifically addressed whether glucose tablet candy intake can mitigate decreases in sustained attention, as measured using an attention test, following smartphone-based cognitive fatigue, with concomitant observation of neurophysiological (EEG) changes during the task. To our knowledge, this is one of the first studies to examine whether glucose tablet candy supplementation can mitigate attention deficits induced by smartphone use.

Ingestion of the glucose tablet candy led to significant improvements in certain sustained attention scores in the post-attention test following smartphone cognitive load and significant attenuation of the decrease in Z-scores for beta wave power at the right prefrontal electrode (placed on the right side of the forehead). Furthermore, VAS ratings demonstrated that physical fatigue and mental clarity (clear-headedness) significantly improved in the post-attention test when the glucose tablet candy was ingested. Collectively, these findings indicate that glucose supplementation may help sustain attention and modulate brain activity after prolonged smartphone use. Attention can be broadly categorized as selective, sustained, or divided attention. Sustained attention is “one of the basic abilities of humans to maintain concentration on relevant information while ignoring irrelevant information over extended periods” [21]. Therefore, the glucose tablet candy may contribute to the enhancement of sustained attention, that is, concentration, which appears to be evident even after a period of smartphone use.

In contrast, VAS scores did not worsen for concentration, physical fatigue, mental fatigue, mental clarity, and motivation, observed in the pre-attention test following the smartphone cognitive load. Significant improvements were observed in concentration, mental clarity, and sleepiness after the load. Collectively, these findings suggest that although participants did not report subjective awareness of fatigue or cognitive decline after the cognitively demanding smartphone cognitive load, ingestion of glucose-based tablet candy was associated with both objective changes in brain activity and improvements in subjective measures, such as physical fatigue and mental clarity. This may ultimately enable individuals to better concentrate on tasks.

Previous studies using the same glucose tablet candy have also demonstrated enhanced sustained attention, in general, cognitive testing [13], as well as similar improvements after esports gameplay [15]. However, the present study extended these findings to a situation where attention deficits were transiently induced by modern smartphone use, a distinct and ecologically relevant form of cognitive load.

Importantly, tasks requiring greater attentional resources and cognitive control, such as sustained attention, heavily depend on frontal regions, particularly the prefrontal cortex [11,12]. This aligns with evidence that the frontal cortex is especially sensitive to energy fluctuations and cognitively demanding tasks are more likely to benefit from glucose facilitation effects.

Our results are consistent with Lim et al. [22], who observed increased beta-wave activity in the frontal Fp1 and Fp2 regions during concentration states. Additionally, beta waves are used in neurofeedback training to enhance attention [17]; the EEG findings of the present study align with these previous reports.

The sustained elevation of beta wave levels in the later part of the post-attention test after smartphone use observed with glucose, but not with the placebo, suggests that glucose supplementation may be associated with beneficial changes in benefit brain activity supporting sustained attention. Notably, while Lim et al. reported increased beta wave activity in both Fp1 (left) and Fp2 (right) during concentration, this phenomenon was only observed at the ChR in the present study. This discrepancy may be attributable to differences in study design; Lim et al. compared different mental states within participants, whereas the present study compared the effects of different foods. This is consistent with a meta-analysis suggesting the existence of a right-lateralized cortical-subcortical network supporting sustained attention [23]. In this study, EEG was analyzed for all recorded frequency bands and across three frontal channels, but only beta power at ChR showed a significant effect of glucose intake.

Notably, in terms of the timing of our measurements relative to blood glucose dynamics, the post-intake attention test in this study was initiated 15 min after glucose ingestion and lasted approximately 20 min. As previously reported in our related studies [13,14], ingestion of the same glucose tablet candy results in a rapid increase in blood glucose concentration, peaking around 30 min and remaining elevated for approximately 60 min before gradually declining. Therefore, our main cognitive and EEG outcomes were assessed during the period of near-maximal glucose availability. This supports the plausibility and timeliness of the observed effects of glucose in the context of acute supplementation.

On the VAS questionnaire administered after the post-attention test, improvements in physical fatigue and mental clarity (clear-headedness) were observed following the ingestion of the glucose tablet candy. This finding may indicate that improvements in the sustained attention test scores were subjectively perceived by the participants. However, although this study aimed to detect negative effects, such as fatigue, induced by the smartphone-based cognitive load task using the VAS questionnaire, the results were different from those after the pre-attention test, where increased fatigue was detected. It is possible that during prolonged smartphone use, individuals may not be fully aware of accumulating fatigue, and, therefore, continue to use their devices. In this study, the cognitive load task was designed to simulate habitual and routine smartphone use in daily life, which may have made the task enjoyable for the participants and consequently made it difficult to detect fatigue or other negative sensations. Therefore, further research is needed to refine the design and evaluation measures for cognitive load tasks to more accurately assess subjective fatigue and related effects.

This study has several limitations. The amount of habitual smartphone use and ways in which smartphones are typically used may have differed among participants, which could have strongly influenced their perception of their smartphone cognitive load. To standardize the cognitive load, we selected tasks on topics that were presumed to be unfamiliar to most participants; however, the two tasks differed in content, and the participants’ interests in the topics likely varied. Therefore, the level of cognitive load may not have been equivalent across sessions. One limitation is that cognitive load was imposed by a 30 min information search on a smartphone, with the load controlled only by task duration rather than by any objective evaluation (such as heart rate variability and salivary cortisol). Therefore, baseline fatigue levels immediately prior to intervention may have varied between conditions. Future studies should more precisely control cognitive load and monitor participants’ fatigue using objective and validated indicators. In comparison, although all participants adhered to the requirement of fasting for at least 4 h before each test day, no upper limit for fasting duration was specified, resulting in minor potential variations in fasting time and prior dietary intake. One important limitation is that EEG signals were recorded solely from three frontal (prefrontal) channels using a simplified instrument, and only the beta frequency band at the right frontal site exhibited a significant glucose effect. This result supports our focus on the beta band in the main analysis; however, future studies should examine the potential roles of other frequency bands and more widespread brain regions using comprehensive EEG measurement. Another limitation is that, although the flavor profiles of both test products were designed to match, subtle differences in sweetness perception between glucose and erythritol may have contributed to subjective responses, which cannot be entirely excluded. It is possible that these factors had some effect on the results, but they were likely to have been less influential than the differences in smartphone use and task conditions.

Despite these limitations, the findings suggest that glucose tablet candy may help counteract attention deficits and related neurophysiological changes associated with smartphone-induced cognitive load. Future work should investigate optimal intervention strategies for real-world cognitive fatigue and further elucidate the neural mechanisms involved.

## 5. Conclusions

In healthy adults aged 18–39 years, the ingestion of a glucose tablet candy (containing 26 g of glucose) led to improvements in sustained attention following smartphone use, accompanied by observable effects on EEG activity. The glucose tablet candy may help to counteract the decline in concentration following cognitively demanding smartphone use.

## Figures and Tables

**Table 1:** Participant characteristics.

Characteristics	Mean ± SD
Age (years)	28.6 ± 7.1
Sex (female/male)	8/8
Height (cm)	165.8 ± 9.7
Body weight (kg)	57.0 ± 9.6
BMI (kg/m^2^)	21.1 ± 2.8
SBP (mmHg)	110.8 ± 14.3
DBP (mmHg)	66.5 ± 12.6

BMI, body mass index; SBP, systolic blood pressure; DBP, diastolic blood pressure.

**Table 2:** Attention test scores.

Task		Test	Standardized Scores		*p*-Values			
			Placebo (*n* = 16)	Glucose (*n* = 16)	Product	Period	Sequence	
ST	Simple reaction time	Pre	97.9 ± 4.5	93.8 ± 1.9				
		Post	92.6 ± 2.6	94.2 ± 2.1	0.263	0.042 *	0.134	
	Complex reaction time	Pre	97.7 ± 3.0	98.8 ± 2.4				
		Post	97.9 ± 2.8	100.0 ± 2.1	0.224	0.003 *	0.625	
	Stroop reaction time	Pre	102.1 ± 3.1	104.1 ± 3.0				
		Post	104.9 ± 2.3	106.2 ± 1.5	0.456	0.057	0.265	
	Stroop commission errors	Pre	94.6 ± 3.0	93.0 ± 4.8				
		Post	89.2 ± 3.4	88.1 ± 5.4	0.788	0.025 *	0.459	
SAT	Correct responses	Pre	108.9 ± 3.0	109.1 ± 2.4				
		Post	112.3 ± 3.3	111.4 ± 3.2	0.732	0.018 *	0.402	
	Errors	Pre	106.8 ± 2.0	106.7 ± 1.7				
		Post	104.1 ± 2.5	103.8 ± 2.7	0.926	0.926	0.282	
	Correct reaction time	Pre	116.6 ± 2.6	117.0 ± 2.6				
		Post	121.2 ± 2.6	119.7 ± 2.4	0.488	0.004 *	0.954	
CPT	Correct responses	Pre	100.3 ± 3.8	102.8 ± 1.3				
		Post	102.1 ± 1.9	100.9 ± 2.2	0.660	0.660	0.087	
	Omission errors	Pre	100.3 ± 3.8	102.8 ± 1.3				
		Post	102.1 ± 1.9	100.9 ± 2.2	0.660	0.660	0.087	
	Commission errors	Pre	99.6 ± 3.8	99.0 ± 2.9				
		Post	104.4 ± 1.7	104.6 ± 2.0	0.959	0.316	0.250	
	Reaction time	Pre	93.2 ± 2.8	94.2 ± 2.2				
		Post	90.5 ± 2.8	92.4 ± 2.9	0.349	0.415	0.063	
FPCPT1	Average correct response time	Pre	102.3 ± 2.0	102.8 ± 1.9				
		Post	104.5 ± 1.4	100.8 ± 2.9	0.123	0.830	0.108	
FPCPT2	Correct responses	Pre	102.5 ± 0.1	102.5 ± 0.1				
		Post	100.8 ± 1.7	102.5 ± 0.1	0.319	0.319	0.319	
	Average correct response time	Pre	96.9 ± 1.7	99.7 ± 3.8				
		Post	95.6 ± 1.9	100.2 ± 1.5	0.039 *	0.878	0.584	
	Incorrect responses	Pre	101.6 ± 2.2	95.8 ± 7.7				
		Post	97.4 ± 4.2	99.3 ± 4.3	0.028 *	0.565	0.353	
	Omission errors	Pre	102.5 ± 0.1	102.5 ± 0.1				
		Post	100.8 ± 1.7	102.5 ± 0.1	0.319	0.319	0.319	
FPCPT3	Correct responses	Pre	109.1 ± 1.9	111.7 ± 0.8				
		Post	111.7 ± 1.0	110.8 ± 1.1	0.179	1.000	0.196	
	Average correct response time	Pre	106.0 ± 2.5	106.7 ± 1.9				
		Post	108.2 ± 2.0	109.3 ± 1.8	0.540	0.243	0.189	
	Incorrect responses	Pre	102.7 ± 0.5	101.8 ± 1.0				
		Post	102.1 ± 0.6	102.4 ± 0.6	0.670	0.570	0.370	
	Omission errors	Pre	109.1 ± 1.9	111.7 ± 0.8				
		Post	111.7 ± 1.0	110.8 ± 1.1	0.179	1.000	0.196	
FPCPT4	Correct responses	Pre	112.5 ± 1.5	109.0 ± 2.5				
		Post	114.3 ± 1.1	112.4 ± 2.2	0.384	0.681	0.505	
	Average correct response time	Pre	105.9 ± 1.8	108.8 ± 1.8				
		Post	109.3 ± 2.0	110.0 ± 1.3	0.757	0.451	0.738	
	Incorrect responses	Pre	104.8 ± 1.9	106.9 ± 1.6				
		Post	106.8 ± 1.3	100.9 ± 4.7	0.237	0.700	0.594	
	Omission errors	Pre	112.5 ± 1.5	109.0 ± 2.5				
		Post	114.3 ± 1.1	112.4 ± 2.2	0.384	0.681	0.505	

Mean ± standard error. ST, Stroop test; SAT, shifting attention test; CPT, continuous performance test; FPCPT, four-part continuous performance test. * *p* < 0.05, corresponding column (Product, Period, or Sequence).

**Table 3:** Effects of test product on visual analog scale (VAS) scores after smartphone cognitive load.

Item	Status	VAS (cm)		*p*-Value		
		Placebo (*n* = 16)	Glucose (*n* = 16)	Product	Period	Sequence
Concentration	Post-load	3.8 ± 0.5	3.0 ± 0.5			
	Post-test	3.8 ± 0.6	3.2 ± 0.6	0.172	0.280	0.562
Physical fatigue	Post-load	4.2 ± 0.4	3.7 ± 0.5			
	Post-test	4.1 ± 0.6	2.9 ± 0.5	0.029 *	0.219	0.403
Mental fatigue	Post-load	4.5 ± 0.4	4.3 ± 0.5			
	Post-test	4.2 ± 0.6	3.3 ± 0.5	0.158	0.754	0.185
Mental clarity (Clear-headedness)	Post-load	4.5 ± 0.4	3.5 ± 0.5			
	Post-test	4.2 ± 0.6	3.1 ± 0.5	0.039 *	0.212	0.454
Motivation	Post-load	4.1 ± 0.5	3.0 ± 0.4			
	Post-test	3.5 ± 0.5	3.2 ± 0.5	0.320	0.932	0.682
Sleepiness	Post-load	2.9 ± 0.4	2.6 ± 0.5			
	Post-test	3.0 ± 0.5	2.2 ± 0.4	0.108	0.386	0.357

Mean ± standard error. * *p* < 0.05, corresponding column (Product, Period, or Sequence). “Post-load” = after smartphone cognitive load; “Post-test” = after post-attention test.

**Table 4:** Effects of smartphone cognitive load on visual analog scale (VAS) scores.

Item	Status	VAS (cm)	*p*-Value		
		(*n* = 32)	Time Points	Period	Sequence
Concentration	At arrival	3.5 ± 0.3			
	Pre-load	4.5 ± 0.3	0.008 *	0.118	0.962
	Post-load	3.4 ± 0.4	0.010 *	0.224	0.462
Physical fatigue	At arrival	2.6 ± 0.3			
	Pre-load	4.0 ± 0.3	0.000 *	0.052	0.613
	Post-load	4.0 ± 0.3	0.949	0.326	0.920
Mental fatigue	At arrival	2.7 ± 0.3			
	Pre-load	4.4 ± 0.3	0.000 *	0.250	0.781
	Post-load	4.4 ± 0.3	0.978	0.859	0.476
Mental clarity (Clear-headedness)	At arrival	3.6 ± 0.3			
	Pre-load	4.8 ± 0.3	0.003 *	0.388	0.645
	Post-load	4.0 ± 0.3	0.043 *	0.068	0.865
Motivation	At arrival	3.2 ± 0.3			
	Pre-load	4.0 ± 0.3	0.050 *	0.550	0.269
	Post-load	3.6 ± 0.3	0.292	0.827	0.528
Sleepiness	At arrival	3.6 ± 0.4			
	Pre-load	4.0 ± 0.4	0.361	0.302	0.182
	Post-load	2.7 ± 0.3	0.003 *	0.027 *	0.722

Mean ± standard error. * *p* < 0.05 in the corresponding column (time points, Period, or Sequence). “At arrival” = on arrival at study site; “Pre-load” = after pre-attention test (before smartphone cognitive load); “Post-load” = after smartphone cognitive load. *p*-values in the “Time points” column indicate the comparison of Pre-load vs. At arrival (in Pre-load status rows) and Post-load vs. Pre-load (in Post-load status rows).

## Data Availability

The data that support the findings of this study are available from the corresponding author upon reasonable request. The data is not publicly available due to privacy concerns.

## Supplementary Materials

The following supporting information can be downloaded at: https://www.mdpi.com/article/10.3390/foods14244233/s1, Figure S1: Power changes (Z-score) in each electroencephalography frequency band (delta, theta, alpha, beta, low gamma, high gamma) at three frontal channels (ChL, ChZ, and ChR) under each condition; Table S1: List of inclusion and exclusion criteria; Table S2: Outline and structure of Cognitrax subtests used in the present study.

## Abbreviations

The following abbreviations are used in this manuscript:

SNS	Social networks
EEG	Electroencephalography
SMR	Sensorimotor rhythm
BMI	Body mass index
SBP	Systolic blood pressure
DBP	Diastolic blood pressure
UMIN	University Hospital Medical Information Network
VAS	Visual analog scale
ST	Stroop test
SAT	Shifting attention test
CPT	Continuous performance test
FPCPT	Four-part continuous performance test
ChZ	Mid-prefrontal electrode
ChR	Right prefrontal electrode
ChL	Left prefrontal electrode
