# Supplementary material for: Effects of Glucose Tablet Candy Ingestion on Attention Following Smartphone Use in Healthy Adults: A Randomized, Double-Blind, Placebo-Controlled Crossover Trial
Source: Foods. 2025 Dec 9;14(24):4233. doi: 10.3390/foods14244233 (PMC12733325; doi:10.3390/foods14244233)
Supplement: Supplementary file 1 [file foods-14-04233-s001.zip › Figure_S1.pdf]

Supplementary Figure

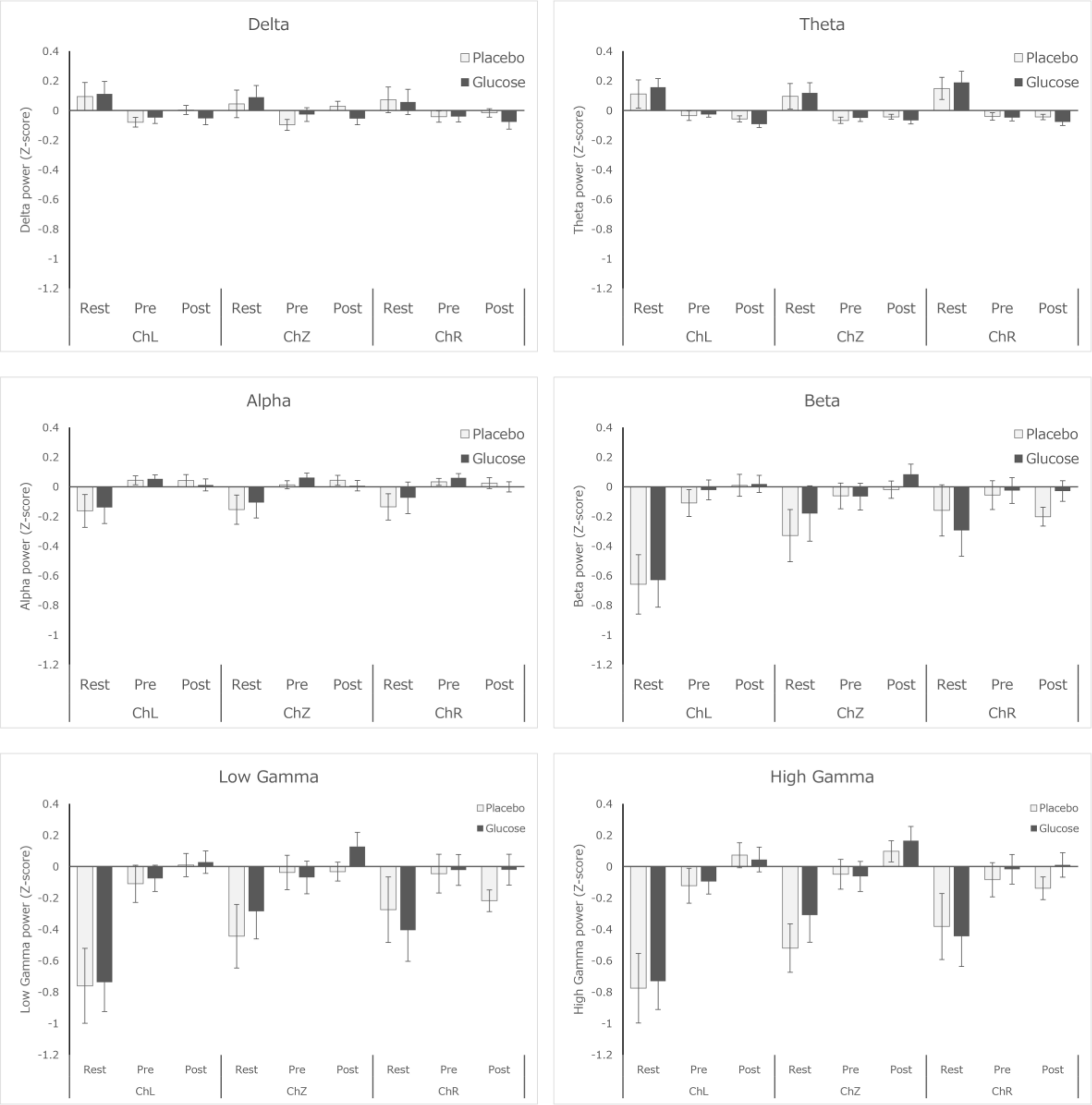

Figure S1. Power changes (Z-score) in each electroencephalography frequency band (delta, theta, alpha, beta, low gamma, high gamma) at three frontal channels (ChL, ChZ, and ChR) under each condition.

Bars indicate mean ± standard error (SE).

ChL, Left prefrontal electrode; ChZ, Mid- prefrontal electrode; ChR, Right prefrontal electrode; Rest, Resting; Pre, Pre-attention test; Post, Post-attention test
