# Supplementary material for: Effects of Glucose Tablet Candy Ingestion on Attention Following Smartphone Use in Healthy Adults: A Randomized, Double-Blind, Placebo-Controlled Crossover Trial
Source: Foods. 2025 Dec 9;14(24):4233. doi: 10.3390/foods14244233 (PMC12733325; doi:10.3390/foods14244233)
Supplement: Supplementary file 1 [file foods-14-04233-s001.zip › Supplementary Tables.pdf]

Supplementary Tables

Table S1. List of inclusion and exclusion criteria

Inclusion criteria
1. Healthy male/female subjects ranging in age from 18 to 39 at informed consent
2. Participants who can give informed consent to participate in this trial after being provided with an explanation of the protocol detail
3. Participants who can visit the designated measurement days to be measured
**Exclusion criteria**
1. Participants who have a habit of continuously taking drugs, foods with health claims (Foods with Nutritional Function Claims, Foods for Specified Health Uses, and Foods with Function Claims), health foods, or supplements that claim to maintain or promote cognitive function (at least three times/week for at least 1 month), currently or within the past 3 months, or those who plan to take them during the study period
2. Participants whose sleeping timeframe substantially fluctuate, such as shift work
3. Participants who may change their lifestyle habits during the study period (changing jobs, moving, long trips, learning, etc.)
4. Participants under another clinical test with medicine or health food, partook in one within the past 4 weeks, or will partake in another clinical test.
5. Participants who may take medicine during the study period due to seasonal allergies (e.g., hay fever)
6. Participants with a smoking habit
7. Participants with excessive alcohol intake
8. Participants with lifestyle diseases (e.g., diabetes mellitus, hypertension, or dyslipidemia) or diagnosed in the past
9. Participants who were diagnosed as having hyperglycemia through a medical examination
10. Participants with neurological disorders, such as dementia, stroke, Parkinson's disease, and epilepsy (excluding headaches and migraines)
11. Participants who received medication for 1 month prior to obtaining consent or plan to receive medication during the study period (excluding history of taking medicine for headache, menstrual pain, cold, etc.)
12. Participants with a past or current history of serious diseases (e.g., heart disease, cancer, or renal failure)
13. Participants who are pregnant, lactating, or planning to become a pregnant during

the study period

14. Participants with sensitive skin to any coatings (e.g., drugs, quasi drugs, or cosmetics), bath salts, tapes, etc., and those with wounds on the head (forehead or near mastoid process [behind ear])
15. Participants with allergies to drug and/or food (including a history of these allergies, especially milk and gelatin)
16. Participants deemed unsuitable for participation in the trial by the principal investigator or sub-investigator

Table S2. Outline and structure of Cognitrax subtests used in the present study.

Task	Part	
ST	1	press the key when a character appears (letters in red, yellow, blue, and green are displayed in black)
	2	press the key when letters and colors match.
	3	press the key when letters and colors do not match.
SAT		Participants followed on-screen instructions and selected the option matching the color or shape. Stimuli combined red, blue, yellow, and green letters, and colors.
CPT		Random letters were displayed one by one. Participants pressed the key only when the letter "B" was displayed.
FPCPT		Shapes: circles, triangles, squares, and stars with colored red, blue, yellow, and green.
	1	press the key when any figure appears.
	2	press the key when a green circle appears.
	3	1-back task
	4	2-back task

ST, Stroop test; SAT, shifting attention test; CPT, continuous performance test; FPCPT, four-part continuous performance test.
